# Total alkaloids of *Leonurus* alleviate allergic asthma and inflammation responses by inhibiting hypoxia-induced factor-1⍺-mediated mast cell activation

**DOI:** 10.3389/fphar.2026.1783196

**Published:** 2026-03-09

**Authors:** Yannan Zheng, Mingyue Lv, Zhichao Xi, Jijia Sun, Mengfan Liu, Trinh Thach Thi Nguyen, Hongmei Zhang, Songlin Li, Min Zheng, Shijie Wan, Hua Li, Man Yuan, Hongxi Xu

**Affiliations:** 1 School of Pharmacy, Shanghai University of Traditional Chinese Medicine, Shanghai, China; 2 Engineering Research Center of Shanghai Colleges for TCM New Drug Discovery, Shanghai, China; 3 College of Artificial Intelligence, Shanghai University of Traditional Chinese Medicine, Shanghai, China; 4 Department of Metabolomics, Jiangsu Province Academy of Traditional Chinese Medicine and Jiangsu Branch of China Academy of Chinese Medical Sciences, Nanjing, China; 5 Department of Chemical and Biomolecular Engineering, University of Pennsylvania, Philadelphia, PA, United States; 6 Fujian Key Laboratory of Chinese Materia Medica, Institute of Structural Pharmacology & TCM Chemical Biology, College of Pharmacy, Fujian University of Traditional Chinese Medicine, Fuzhou, China

**Keywords:** allergic asthma, hypoxia, Leonurus japonicus houtt., mast cell, total alkaloids

## Abstract

**Background:**

*Leonurus japonicus* Houtt. has been traditionally used as a gynecological remedy and is now recognized for its anti-inflammatory and immunomodulatory properties. But its potential therapeutic role in allergic asthma is unclear. This study aimed to investigate the anti-asthmatic effects of the total alkaloids of Leonurus (TAL) and their regulation of mast cell activation through the hypoxia-inducible factor-1α (HIF-1α) pathway.

**Materials and Methods:**

An ovalbumin (OVA)-induced asthma mouse model was used to detect TAL’s effects on airway hyperresponsiveness, lung inflammation, and Th2 cytokines. For *in vitro* studies, RBL-2H3 cells were employed as a mast cell model. Transcriptomics and cross-species analysis integrated rat signatures with human asthma datasets using KEGG enrichment to identify conserved HIF-1α–related pathways. HIF-1α expression and localization were analyzed by Western blotting and immunofluorescence. Mast cell degranulation was evaluated *via* β-hexosaminidase (β-hex) assay and Th2 cytokines detection. Constructed a HIF-1α stable overexpression cell or applied pharmacological inhibition (YC-1) to verify the key role of HIF-1⍺ on TAL’s efficacy.

**Results:**

TAL significantly alleviated allergic symptoms, reduced airway hyperresponsiveness, and decreased lung mast cell infiltration. Transcriptomic analysis revealed that hypoxia-related pathways, particularly HIF-1α, were downregulated following TAL intervention. TAL suppressed HIF-1α protein expression, reduced mast cell degranulation, and Th2 cytokines both *in vivo* and *in vitro*. Mechanistically, hypoxia induced HIF-1α upregulation, mast cell activation, and cytokine release, whereas TAL inhibited these responses. This effect was weakened by HIF-1α overexpression but potentiated by YC-1.

**Conclusion:**

TAL alleviates allergic asthma by suppressing HIF-1α-mediated mast cell activation and associated inflammation responses, supporting its potential application in inflammatory airway diseases.

## 1 Introduction

Asthma is a chronic inflammatory airway condition impacting approximately 300 million people globally, with increasing incidence attributed to environmental and genetic influences (Dharmage et al., 2019). Allergic asthma, affecting approximately 60% of adults and 80%–90% of children with asthma (Papapostolou and Makris, 2022), is recognized by current Global Initiative for Asthma (GINA) guidelines as the most common subtype. It is primarily characterized by reversible respiratory obstruction, mucus hypersecretion and airway hyperresponsiveness. Current treatments primarily include inhaled corticosteroids and β_2-_agonists, often combined with leukotriene receptor antagonists or biologics targeting IgE or interleukins (Holgate et al., 2015). However, these therapies can be ineffective in some patients, which is steroid-resistant, severe-refractory, or biologic-unresponsive, have limited capacity to induce disease remission, and may cause adverse effects with long-term use, highlighting the need for novel therapeutic strategies.

Multiple immune cell types contribute to asthma pathophysiology, among which mast cells play a pivotal role. These granule-rich cells, abundant in the airway mucosa, are rapidly activated upon allergen exposure, releasing β-hexosaminidase (β-hex), histamine, leukotrienes and proinflammatory cytokines. These mediators sustain Th2-dominant immune responses, exacerbate bronchoconstriction, and promote airway remodeling (Bradding et al., 2006; Elieh Ali Komi and Bjermer, 2019). Given their central role in disease progression, targeting mast cell activation and Th2-mediated immunity represents a promising therapeutic strategy.

Recent studies have emphasized the critical role of the tissue microenvironment and particularly hypoxia in modulating immune responses in asthma. Hypoxia is a hallmark of inflamed airway tissues and contributes to persistent inflammation and structural remodeling (Mikami et al., 2023). Hypoxic conditions have been shown to increase the production of proinflammatory mediators, featuring VEGF, IL-4, IL-5, and IL-13, in bone marrow-derived mast cells, hence intensifying allergic responses (Lee et al., 2008). Hypoxia-inducible factor-1α (HIF-1α), a principal transcriptional regulator of cellular adaptation to hypoxia, has emerged as a key mediator in asthma pathophysiology (Wan et al., 2024). Experimental models indicate that elevated HIF-1α expression in lungs with asthma corresponds with disease severity and airway remodeling. (Zhang et al., 2025). Moreover, HIF-1α activation influences mast cell degranulation by modulating critical signaling pathways including PI3K-Akt, NF-κB, and MAPKs, thereby amplifying inflammations within the hypoxic microenvironment (Lee, et al., 2008; Zhang et al., 2021; Zhao et al., 2020). Given its central role in sustaining airway inflammation, HIF-1α is a prospective treatment target for asthma. Nevertheless, the precise mechanisms by which HIF-1α regulates mast cell activation and Th2-driven immune responses remain to be fully understood.

From the perspective of traditional medicine, natural products have long served as a valuable source for discovering novel anti-inflammatory agents. *Leonurus japonicus* Houtt., commonly known as motherwort, is traditionally used in gynecological diseases. (Shang et al., 2014). Phytochemical investigations have revealed that motherwort contains various bioactive constituents, including alkaloids, flavonoids, iridoids and diterpenoids, among which the total alkaloids fraction represents one of the major active fractions (Shang, et al., 2014). The total alkaloids of *Leonurus* (TAL) have demonstrated significant pharmacological activities, including anti-inflammatory, pro-angiogenic and immunoregulatory effects (Angeloni et al., 2021; Dai et al., 2025; Shi et al., 2022). Previous researchers suggest that TAL can modulate inflammatory pathways by inhibiting pro-inflammatory cytokines and oxidative stress. However, its potential application in asthma treatment is still underexplored.

In this study, we demonstrated that TAL effectively alleviated allergic symptoms in a murine ovalbumin (OVA)-induced allergic asthma model and inhibited RBL-2H3 cells activation. Further mechanistic analysis reveals that TAL’s anti-asthmatic effects are potentially mediated through suppression of HIF-1α-driven mast cell reactivation and associated inflammation. Our findings provide pharmacological evidence supporting the ethnomedicinal use of *L. japonicus* and identify HIF-1α as a promising target for asthma intervention.

## 2 Materials and Methods

### 2.1 Drugs and reagents

The extract of *L. japonicus* Houtt., referred to as the total alkaloids of *Leonurus* (TAL), was supplied by GKH Pharmaceutical Ltd. (Guangzhou, China). TAL was prepared according to a patented extraction and purification procedure (Chinese Patent No. CN02117432.6). Briefly, dried aerial parts of *L*. japonicus were subjected to aqueous extraction followed by ethanol precipitation. The crude extract was subsequently purified using strong and weak acid cation-exchange resins to enrich the total alkaloid fraction. Batch consistency and chemical fingerprinting of TAL were previously characterized using UHPLC-Q-TOF-MS in our earlier study (Shi, et al., 2022), ensuring the reproducibility and quality stability of the extract used in the present experiments. The resulting preparation contained more than 50% total alkaloids (w/w). The major identified alkaloid constituents included stachydrine, trigonelline, choline, and leonurine. We bought dinitrophenyl (DNP)-IgE, OVA, cobaltous chloride (CoCl_2_), and methacholine from Sigma-Aldrich (MO, United States). Alum adjuvant was purchased from Thermo Fisher Scientific Inc. (Waltham, United States). DNP-human serum albumin (HSA) protein conjugate was purchased from Alpha Diagnostic Inc. (San Antonio, United States). Tribromoethanol was bought from Nanjing Aibei Biotechnology Co., Ltd. (Nanjing, China). The specific primary antibodies against HIF-1⍺, β-actin and GAPDH were bought from Cell Signaling Technology (Boston, United States). We purchased the HIF-1⍺ inhibitor, Lificiguat (YC-1), from Selleck Chemicals (Houston, United States).

### 2.2 Animals

BALB/c female mice, with body weights ranging from 18 to 22 g, were obtained from the Shanghai SLAC Laboratory (Shanghai, China). The animals were maintained under specific pathogen-free conditions, with controlled temperature (22 °C ± 1 °C), relative humidity (55% ± 5%), and a 12 h light/dark cycle. All procedures complied with institutional ethical standards and were approved by the Ethics Committee of Shanghai University of Traditional Chinese Medicine (Approved No. PZSHUTCM2507170006).

### 2.3 OVA-induced allergic asthma model and TAL treatment

**FIGURE 1:**
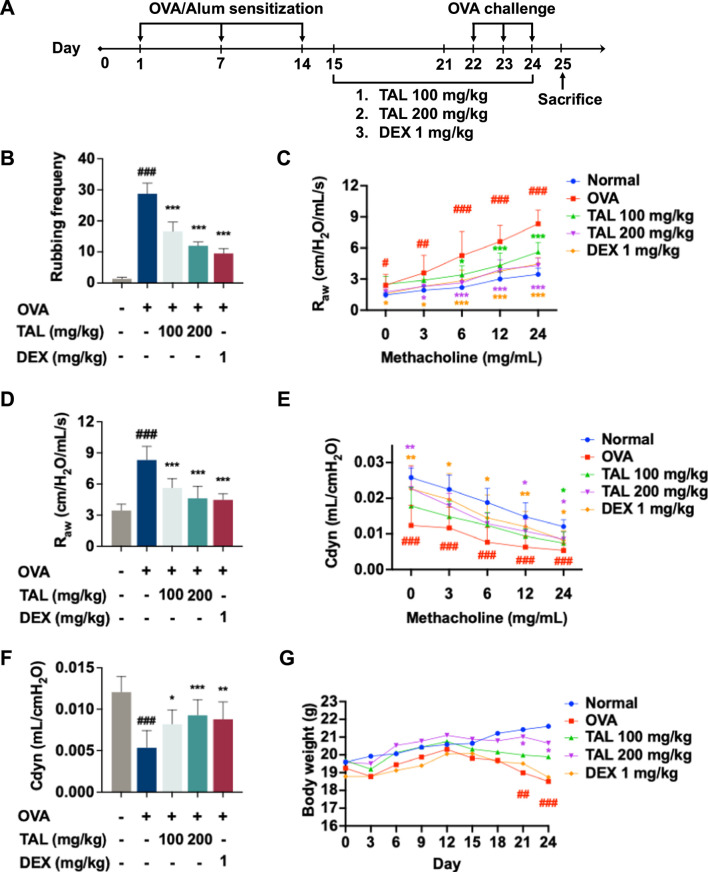
TAL alleviates allergic symptoms and airway hyperresponsiveness (AHR) in the OVA-induced asthma model. **(A)** Schematic diagram of the experimental protocol. **(B)** Nasal rubbing frequency following the final OVA challenge during the 15 min. **(C)** Airway resistance (R_aw_) following increasing doses of methacholine. **(D)** R_aw_ at a concentration of 24 mg/mL of methacholine. **(E)** Dynamic compliance (Cdyn) following increasing doses of methacholine. **(F)** Cdyn at a concentration of 24 mg/mL of methacholine. **(G)** Change in body weight. Data are expressed as mean ± SEM (n = 8). ^#^ *P* < 0.05, ^##^ *P* < 0.01, ^###^ *P* < 0.001 compared with the normal group. ^*^ *P* < 0.05, ^**^ *P* < 0.01, ^***^ *P* < 0.001 compared with the OVA group.

Forty female BALB/c mice were divided into the following five groups (eight mice per group): a normal control, an OVA model, a low-dose TAL (100 mg/kg), a high-dose TAL 200 mg/kg), and a dexamethasone (DEX 1 mg/kg) treatment group. Following a 1-week adaptation, all mice except those in the normal group underwent sensitization, which was performed by intraperitoneal delivery of 200 μL OVA-alum solution (40 µg OVA dissolved in alum adjuvant and PBS) on days 0, 7 and 14. From days 15–21, mice were orally administered saline, TAL or DEX once daily. Subsequently, asthma was induced *via* aerosol exposure to 5% OVA (in PBS) for 30 min per day over 3 consecutive days (day 22–24). Animals were anesthetized with tribromoethanol and euthanized on day 25. Blood was collected *via* cardiac puncture for serum isolation, and lung and spleen tissues were harvested for subsequent experiments. The schematic representation of the OVA-induced allergic asthma model, along with the TAL treatment, is shown in Figure 1A. The dose range was selected according to previously reported pharmacological studies and adjusted based on preliminary experiments to ensure efficacy and safety.

### 2.4 Measurement of airway hyperresponsiveness

Airway hyperresponsiveness was invasively assessed using the FinePointe™ Resistance and Compliance System (Buxco RC, DSI, United States). Mice were anesthetized with 1.25% tribromoethanol and mechanically ventilated, followed by aerosolized delivery of methacholine at increasing concentrations (0, 3, 6, 12 and 24 mg/mL in PBS). The protocol consisted of a 30 s acclimatization phase, a 20 μL aerosol administration over 30 s, a 3 min response period, and a 30 s recovery phase for each concentration. Airway resistance (R_aw_) and dynamic compliance (Cdyn) were recorded to evaluate pulmonary function.

### 2.5 Histological analysis

The left lung lobes were inflated and fixed in 4% paraformaldehyde, followed by dehydration, clearing, paraffin embedding and sectioning. The subsequent step involved the staining of tissue slices with hematoxylin and eosin (H&E; Beyotime, Shanghai, China) or toluidine blue (TB; Ruiyuan, Shanghai, China) following the method of Lertnimitphum (Lertnimitphun et al., 2021). Microscopic observations were performed with an Olympus DP-72 microscope (DP-72, Tokyo, Japan).

### 2.6 Cytokines enzyme-linked immunosorbent assay (ELISA)

After the mice were sacrificed, the cardiac blood was obtained and maintained at 4 °C for 4–6 h and then centrifugation. The left pulmonary lobe of each mouse was excised and rapidly snap-frozen in liquid nitrogen. Spleens were collected from mice, and spleen cells were stimulated using OVA for 3 days. Serum IgE and Th2 cytokines (IL-4 and IL-13) in lung homogenates and splenocyte lysates were quantified using ELISA kits in accordance with the manufacturer’s protocols. For measuring IL-4 and IL-13 levels *in vitro*, mast cells were cultured under the indicated treatment conditions. After stimulation, culture supernatants were harvested, clarified by centrifugation, and cytokines were determined using ELISA kits according to the supplier’s guidelines. Mouse IgE (Catalogue number: EMC117), IL-4 (Catalogue number: EMC003) and IL-13 (Catalogue number: EMC124) ELISA kits were obtained from Neobioscience (Shenzhen, China). Rat IL-4 ELISA kit (Catalogue number: RK00040) was supplied from ABclonal Biotechnology Co., Ltd. (Wuhan, China). Rat IL-13 ELISA kit (Catalogue number: RX302875R) was purchased from Ruixin Biotech (Quanzhou, China).

### 2.7 Bioinformatics analysis

The study retrieved microarray data (GSE76262) from the GEO public database. This data comprised samples from healthy controls and patients with severe asthma (SA). The raw data was then subjected to a series of preprocessing steps, including background correction and quantile normalisation, utilising the Robust Multi-array Average (RMA) algorithm. Differentially expressed genes (DEGs) were determined with the limma package in R, under the criteria of adjusted *P* < 0.05 and |log_2_ fold change| > 1. Weighted Gene Co-expression Network Analysis (WGCNA) was applied to identify gene modules related to SA. A soft-threshold power of β = 10 was chosen to approximate a scale-free topology. Modules were generated by dynamic tree cutting, and correlations between modules and traits were calculated to screen asthma-associated modules. Genes overlapping between key modules and DEGs were considered candidate disease-related genes. Protein–protein interaction (PPI) networks for overlapping genes were established through the STRING database with an interaction score >0.4 and further visualized with Cytoscape v3.9.1. Receiver operating characteristic (ROC) curve analysis was carried out with the pROC package to evaluate the diagnostic potential of candidate genes, such as HIF-1⍺. Gene set enrichment analysis (GSEA) was performed using the cluster Profiler package, based on MSigDB Hallmark and KEGG gene sets. Pathways meeting the thresholds of FDR <0.25 and *P*-value <0.05 were considered statistically significant.

### 2.8 Cell culture and hypoxia model

RBL-2H3 cells (Wistar rat-derived basophilic leukemia cell line used in Mast cell research) were bought from Pricella Biotechnology Co., Ltd. (Wuhan, China). Cells were cultured using MEM medium (Meilunbio, China) supplemented with 15% FBS (Biosharp, China) and 1% penicillin-streptomycin (Meilunbio, China) in a humidified incubator at 37 °C with 5% CO_2_. Chemical hypoxia was induced by treating the cells with CoCl_2_ (dissolved in DMSO) at 100 μM and 6 h.

### 2.9 CCK-8 assay

The cytotoxicity of TAL was evaluated using a CCK-8 assay. RBL-2H3 cells (1 × 10^4^ cells per well) were seeded into 96-well plates and incubated for 24 h. Cells were then treated with TAL (0–4 mg/mL) for 24 h. Subsequently, the culture medium was replaced with fresh medium containing 10% CCK-8 reagent, and the plates were incubated at 37 °C for 1.5 h. The absorbance (OD) of each well was measured at 490 nm using a microplate reader (Agilent, Santa Clara, United States).

### 2.10 Detection of mast cell degranulation

Mast cell degranulation was assessed by measuring the release of β-hex. The specific procedure was as follows: (1) RBL-2H3 cells (2 × 10^4^ cells per well) were seeded in 24-well plates and pre-sensitized overnight with 500 ng/mL DNP-IgE in MEM medium. (2) On the following day, cells were washed with PBS and treated with TAL (0, 0.5, 1.0, 2.0 and 4.0 mg/mL) or YC-1 (20 µM) for 2 h (3) The supernatant was then discarded, and the cells were stimulated with 100 ng/mL DNP-HSA for 1 h, excluding the vehicle control group. The reaction was terminated by cooling at 4 °C for 10 min, after which the supernatant was collected for β-hex activity assay. For the assay, the details were performed as previously described (Zhang, et al., 2021). Optical density was recorded at 405 nm using a microplate reader. The percentage of β-hex release was calculated according to the formula: β-hex release (%) = (OD_TAL_–OD_vehicle_)/(OD_DNP-HSA_–OD_vehicle_) ×100.

### 2.11 Lentivirus infection

RBL-2H3 cells were seeded in 6-well plates at an initial density of 5 × 10^5^ cells per well. Cells were transduced with lentivirus carrying either pLV-CB-Hif1a-P2A-PGK-Puro (RBL-2H3 HIF-1α) or pLV-CB-PGK-Puro (EV). After 48 h, the culture medium was replaced with fresh complete medium. Following a 72-h period of post-transduction, puromycin (1 μg/mL) was introduced to initiate the selection process. The selection medium was refreshed every 2–3 days until all non-transduced cells died. Subsequently, HIF-1α expression was validated at the mRNA and protein levels by RT-PCR and Western blotting respectively. The lentivirus overexpressing HIF-1α were constructed and packaged by Shanghai Bio-lifespan Co., Ltd. (Shanghai, China).

### 2.12 Western blotting

Western blotting procedure was followed previous published study (Chang et al., 2025). Primary antibodies against anti-HIF-1α (1:1000, Cell Signaling Technology, 36169S), anti-β-actin (1:1000, Cell Signaling Technology, 4967S), and anti-GAPDH (1:1000, 2118S) followed by HRP-conjugated secondary antibodies (1:2500). Protein bands were visualized with an Amersham ImageQuant LAS 800 system (Cytiva, Wilmington, United States) and quantified with ImageJ software.

### 2.13 Reverse transcription polymerase chain reaction (RT-PCR)

Before establishing the stably transfected cell line, RT-PCR detection was performed as previously described (Xi et al., 2025). The primer sequences were as follows: HIF-1α forward, 5′-GTG​ACC​GTG​CCC​CTA​CTA​TG-3′, HIF-1α reverse, 5′-CGT​AAC​TGG​TCA​GCT​GTG​GT-3’.

### 2.14 Statistical analysis

Statistical analyses in this study were performed with GraphPad Prism 9. Differences between the two groups were evaluated using an unpaired Student’s t-test, and *ANOVA* was applied for comparisons involving more than two groups. The data are presented as mean ± SD from at least three independent experiments. The statistical significance of *P* was determined by employing a *P*-value cut-off of <0.05.

## 3 Results

### 3.1 TAL alleviates airway hyperresponsiveness (AHR) in OVA-induced asthmatic mice

A model of asthma, induced by OVA, was established in accordance with protocols that had previously been described, with the purpose of evaluating the anti-asthmatic effects of TAL, as illustrated in Figure 1A. Both TAL and DEX treatments significantly reduced nasal rubbing behavior after the final OVA challenge compared to the OVA model group, a characteristic of the immediate allergic response, indicating effective relief of allergic symptoms (Figure 1B). AHR, a hallmark of asthma, was assessed by measuring R_aw_ and Cdyn in response to rising methacholine (0–24 mg/mL). Asthmatic mice exhibited a significant increase in R_aw_ and a decrease in Cdyn, confirming successful induction of AHR (Figures 1C,E). TAL treatment effectively attenuated methacholine-induced elevation in R_aw_ and partially restored Cdyn. At the highest methacholine concentration (24 mg/mL), TAL markedly decreased R_aw_ and increased Cdyn compared to the asthma model group (Figures 1D,F), with the high-dose TAL group showing efficacy comparable to DEX. Additionally, asthmatic mice experienced progressive body weight loss during the OVA challenge phase, which was significantly alleviated by TAL treatment, especially at the lower dosage (100 mg/kg), whereas DEX exhibited no significant effect on body weight (Figure 1G). These results demonstrate that TAL effectively ameliorates allergic symptoms and improves lung function in OVA-induced asthmatic mice.

### 3.2 TAL attenuates OVA-induced allergic inflammation and mast cell infiltration

**FIGURE 2:**
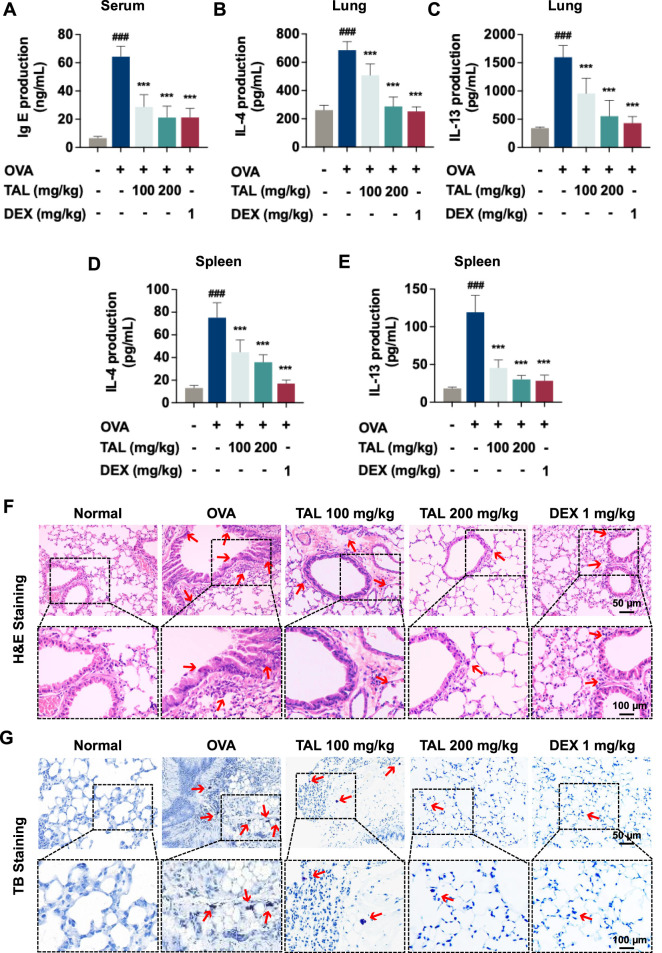
TAL reduces OVA-induced allergic inflammation and mast cell activation. **(A)** Serum total IgE levels. **(B,C)** Th2 cytokine levels in lung tissue homogenates (IL-4 **(B)** and IL-13 **(C)**). **(D,E)** Th2 cytokine secretion from splenocytes collected from mice and stimulated with OVA for 72 h (IL-4 **(D)** and IL-13 **(E)**). **(F)** H&E staining of lung sections (scale bar: 50 μm and 100 μm). **(G)** TB staining of lung sections (scale bar: 50 μm and 100 μm). Data are expressed as mean ± SEM (n = 8). ^###^ *P* < 0.001 compared with the normal group. ^***^ *P* < 0.001 compared with the OVA group.

To assess the effects of TAL on respiratory inflammatory response, serum IgE levels were measured in asthmatic mice. IgE levels were significantly elevated in the OVA group compared with the normal group and were markedly reduced by TAL in a dose-dependent manner, with the high dose showing the strongest effect (Figure 2A). In lung tissue homogenates, the Th2 cytokines IL-4 and IL-13 were increased in the OVA group and significantly suppressed by TAL treatment (Figures 2B,C). Similarly, splenocytes from asthmatic mice secreted higher levels of IL-4 and IL-13 upon OVA stimulation, which were reduced by TAL in both dose groups (Figures 2D,E). DEX, used as a positive control, significantly decreased serum IgE, as well as the levels of both IL-4 and IL-13 in lung tissue and splenocyte cultures.

Moreover, allergic asthma is characterized by eosinophil-dominated inflammatory infiltration and mast cell activation. H&E staining of lung sections revealed extensive inflammatory cell infiltration and airway remodeling in the OVA group (Figure 2F). TAL-treatment markedly reduced inflammatory infiltration in the lung tissues compared with the asthmatic model group. Mast cell activation, assessed by TB staining, showed a significant increase in mast cell numbers and degranulation in the lung tissues of the OVA group (Figure 2G). Both low- and high-dose TAL treatment significantly reduced the number of activated mast cells, with high-dose treatment showing the most pronounced effect. These findings indicate that TAL mitigates systemic and local allergic inflammation in asthmatic mice by reducing IgE production, suppressing Th2 cytokine expression, and alleviating eosinophil infiltration and mast cell activation in the lungs.

### 3.3 TAL modulates hypoxia-associated pathways

Hypoxia is a well-established feature of asthma that drives airway inflammation and immune dysregulation through hypoxia-inducible pathways. To investigate the potential inhibitory effect of TAL under hypoxic conditions, we employed a chemical hypoxia model in RBL-2H3 mast cells using CoCl_2_. Cytotoxicity of TAL was assessed by CCK-8 assay, revealing no significant toxicity at concentrations from 0.125 to 2 mg/mL, while 4 mg/mL significantly reduced cell viability (Supplementary Figure S1). Consequently, 2 mg/mL was chosen as the maximum concentration for further *in vitro* investigation to maintain biological relevance without affecting cell viability.

**FIGURE 3:**
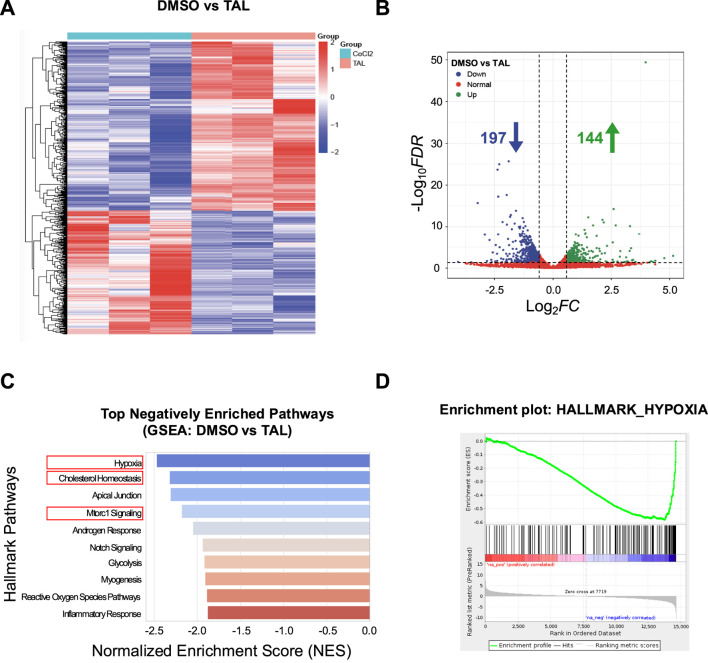
TAL reverses hypoxia-associated signaling in mast cells. **(A)** Heatmap showing gene expression profiles in DMSO and TAL groups (n = 3). **(B)** Volcano plot of differentially expressed genes between groups (adjusted p < 0.05, |log_2_FC| > 1). **(C)** Top 10 negatively enriched hallmark pathways identified by GSEA comparing DMSO vs. TAL. **(D)** GSEA enrichment plot of the Hypoxia gene set.

To further investigate the mechanism by which TAL alleviates asthma, RNA sequencing was conducted to compare the transcriptomic profiles of the DMSO control and TAL-treated samples in the CoCl_2_-induced hypoxia model, with three biological replicates per group. The RNA sequencing data have been deposited in the NCBI Sequence Read Archive (SRA) under BioProject accession number PRJNA1426373. As shown in Figure 3A, a clear separation in gene expression patterns between the two groups was observed. The TAL group was found to have 341 differentially expressed genes (DEGs), of which 144 were found to be overexpressed and 197 underexpressed (Figure 3B). GSEA based on the ranked gene list showed significant downregulation of hallmark hypoxia-related pathways in the TAL group, such as hypoxia, cholesterol homeostasis, and mTORC1 signaling supporting the notion that TAL mitigates CoCl_2_-induced hypoxia (Figures 3C,D).

### 3.4 HIF-1α is identified as a key regulator in TAL’s anti-asthma effects and a potential diagnostic indicator in asthma *via* cross-species transcriptomics

**FIGURE 4:**
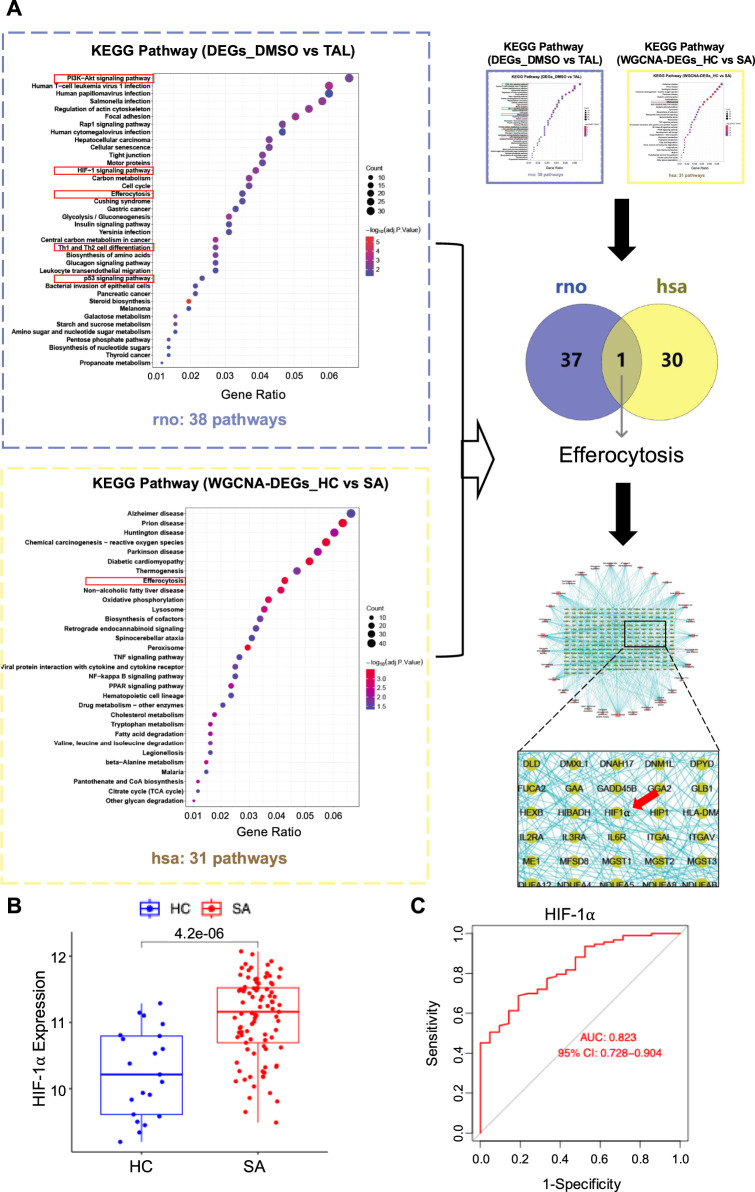
HIF-1α is involved in the mechanism underlying TAL’s anti-asthma effects. **(A)** Schematic diagram of the cross-species transcriptomics. KEGG enrichment analysis of DEGs between DMSO- and TAL-groups (rno). KEGG enrichment analysis (hsa) of intersecting genes derived from DEGs and severe asthma (SA)-associated modules identified by WGCNA. Transcriptomic data were obtained from the GSE76262 clinical dataset. Venn diagram showing intersecting KEGG pathways between rat and human datasets. PPI network based on intersecting genes. **(B)** Boxplot showing HIF-1α expression levels in healthy controls (HC) versus SA patients. **(C)** ROC curve analysis of HIF-1⍺ in the GSE76262 dataset, evaluating its diagnostic performance in severe asthma. Transcriptomic data were obtained from the GSE76262 clinical dataset.

To identify the key hypoxia-related regulator in asthma, we performed a cross-species integrative analysis (Figure 4A). KEGG pathway enrichment analysis of CoCl_2_-induced hypoxic RBL-2H3 cells compared with TAL-treated cells revealed 38 significantly altered pathways. To assess the clinical relevance of hypoxia-associated pathways in asthma, we analyzed transcriptomic profiles from healthy controls (HC, n = 21) and severe asthma (SA, n = 118) patients, in the publicly available GSE76262 dataset (GEO) using WGCNA, identifying 30 significantly enriched KEGG pathways (Supplementary Figure S2). A Venn diagram comparing enriched KEGG pathways from both human (hsa) and rat (rno) datasets revealed a single shared pathway—efferocytosis (Figure 4A), highlighting its conserved relevance across the *in vitro* mast cell model and human allergic asthma pathology. A PPI network constructed from efferocytosis-related DEGs identified HIF-1α as a central hub, underscoring its role of HIF-1α-regulated efferocytosis in asthma (Figure 4A).

Boxplot analysis showed significantly elevated HIF-1α expression in SA patients compared with HC, supporting its potential role as a diagnostic indicator (Figure 4B). ROC analysis further demonstrated a favorable area under the curve (AUC), indicating strong discriminatory value for SA diagnosis (Figure 4C). Collectively, these findings not only reinforce the pivotal role of HIF-1α in asthma pathogenesis but also suggest its involvement in the mechanism underlying TAL’s anti-asthma effects.

### 3.5 TAL inhibits HIF-1α signaling, suppresses mast cell activation and attenuates associated inflammatory responses

**FIGURE 5:**
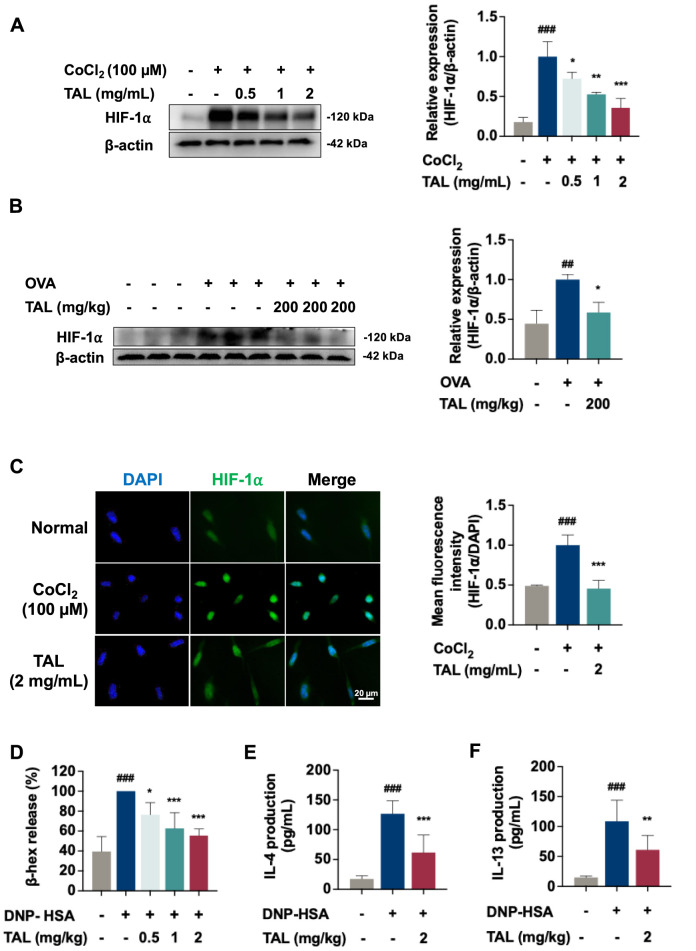
TAL inhibits HIF-1α expression and mast cell activation. **(A)** HIF-1α protein expression in RBL-2H3 cells under CoCl_2_ (100 μM) induced-hypoxic with or without TAL treatment (0.5–2 mg/mL). **(B)** HIF-1α protein levels in lung tissues. **(C)** Immunofluorescence images and quantification of mean fluorescence intensity of HIF-1α in RBL-2H3 cells under hypoxic with or without TAL treatment (2 mg/mL). **(D)** β-hex release in RBL-2H3 cells following DNP-HSA stimulation and TAL treatment (0.5–2 mg/mL). **(E,F)** Levels of IL-4 **(E)** and IL-13 **(F)** secretion in RBL-2H3 cells following DNP-HSA stimulation and TAL treatment. Data are presented as mean ± SEM. from three independent experiments. ^##^ *P* < 0.01, ^###^ *P* < 0.001 compared with the normal group. ^*^ *P* < 0.05, ^**^ *P* < 0.01, ^***^ *P* < 0.001 compared with the OVA or model group.

Our transcriptomic analysis identified HIF-1α signaling as one of the pathways most significantly downregulated by TAL, suggesting that inhibition of this pathway could contribute to its anti-asthma effects. To validate this prediction, we examined whether TAL inhibited HIF-1α activation. In RBL-2H3 cells, CoCl_2_-induced chemical hypoxia markedly increased HIF-1α protein levels. TAL treatment (0.5–2 mg/mL) significantly reduced HIF-1α expression in a dose-dependent manner (Figure 5A). Similarly, lung tissues from OVA-induced asthmatic mice exhibited elevated HIF-1α protein levels compared with the normal control group. Whereas oral TAL administration (200 mg/kg) significantly decreased pulmonary HIF-1α expression (Figure 5B).

Nuclear translocation of HIF-1α is a pivotal step in activating hypoxia-responsive transcriptional programs, which in mast cells drive downstream inflammatory signaling, degranulation, and the release of pro-inflammatory mediators (Walczak et al., 2008). Under hypoxic conditions, mast cells displayed markedly increased HIF-1α fluorescence intensity, predominantly localized within the nucleus. TAL treatment not only reduced overall HIF-1α expression but also diminished its nuclear accumulation, indicating inhibition of HIF-1α activation and transcriptional activity (Figure 5C). To assess the functional consequences of this modulation, we examined whether TAL attenuated mast cell degranulation induced by DNP-HSA. TAL significantly inhibited β-hex release from RBL-2H3 cells in a dose-dependent manner, indicating reduced degranulation activity (Figure 5D). Moreover, TAL markedly suppressed the secretion of key Th2 cytokines, IL-4 and IL-13, both of which were elevated following DNP-HSA stimulation (Figures 5E,F). Collectively, these findings confirm the transcriptomic prediction that TAL inhibits HIF-1α signaling and demonstrate that TAL mitigates hypoxia-induced HIF-1α upregulation and suppress mast cell activation.

### 3.6 Overexpression of HIF-1α promotes mast cell activation and attenuates TAL’s inhibitory effect

**FIGURE 6:**
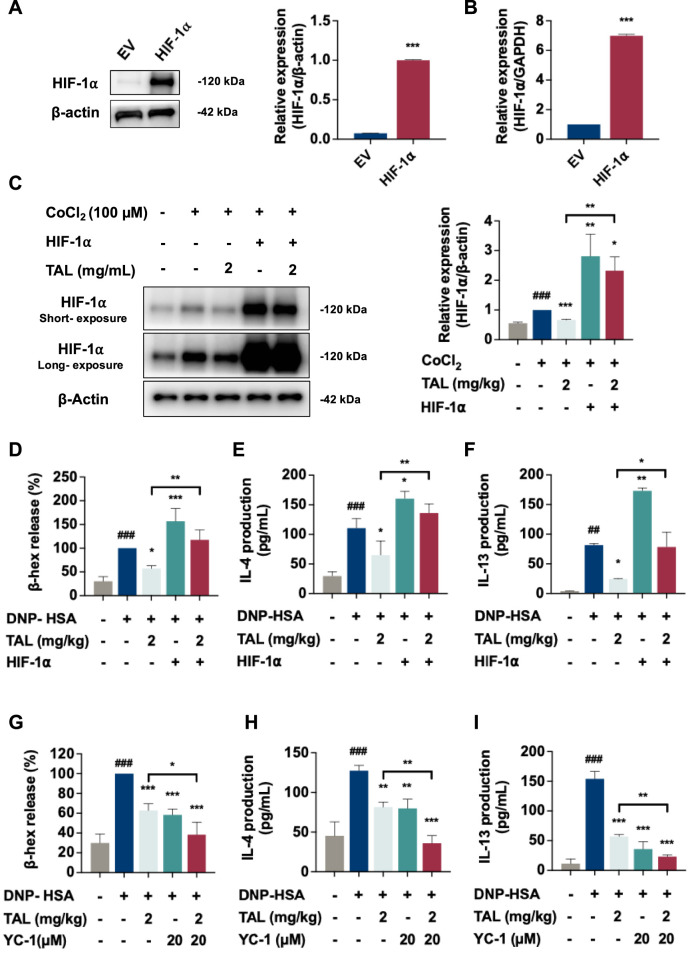
HIF-1α promotes mast cell activation and attenuates TAL’s inhibitory effect. **(A,B)** Western blot **(A)** and RT-PCR **(B)** validation of the levels of HIF-1α in RBL-2H3 cells. **(C)** Western blot analysis of HIF-1α protein expression in EV and HIF-1α-overexpressing cells with or without TAL treatment. **(D–F)** Levels of β-hex **(D)**, IL-4 **(E)**, and IL-13 **(F)** in the indicated groups following DNP-HSA stimulation and TAL treatment. **(G–I)** Effect of the HIF-1α inhibitor YC-1 (20 µM) on β-hex release **(G)**, IL-4 **(H)**, and IL-13 **(I)** secretion in DNP-HSA-stimulated RBL-2H3 cells. Data are expressed as mean ± SEM from three independent experiments. ^##^ *P* < 0.01, ^###^ *P* < 0.001 compared with the control. ^*^ *P* < 0.05, ^**^ *P* < 0.01, ^***^ *P* < 0.001compared with the DNP-HSA simulated group or as indicated.

To determine whether HIF-1α serves as a key regulator of TAL-mediated inhibition of mast cell activation, we established a stable HIF-1α-overexpressing RBL-2H3 cell line (RBL-2H3 HIF-1α) by transfecting cells with a full-length HIF-1α cDNA construct or an empty vector (EV) as a control. Overexpression was confirmed by elevated protein (Figure 6A) and mRNA levels (Figure 6B). In EV control cells, hypoxic conditions induced by CoCl_2_ markedly increased HIF-1α protein expression, which was then significantly reduced by TAL treatment. In contrast, in HIF-1α-overexpressing cells, CoCl_2_-induced HIF-1α expression remained substantially elevated, diminishing TAL’s ability to reduce HIF-1α levels. Compared with the hypoxic model group, HIF-1α levels in the overexpression group remained significantly higher and were also markedly elevated compared with TAL-treated EV cells under CoCl_2_ (Figure 6C). These results indicate that HIF-1α overexpression partially impairs TAL’s ability to regulate HIF-1α expression under hypoxic conditions.

Functionally, we next examined whether overexpression affected TAL’s inhibitory effects on mast cell degranulation and cytokine release. In EV control cells, DNP-HSA stimulation markedly increased β-hex release, which was significantly suppressed by TAL. However, HIF-1α overexpression further enhanced β-hex release compared with the EV control cells, and significantly reversed TAL’s inhibitory effect (Figure 6D). Similarly, IL-4 and IL-13 secretion were upregulated upon DNP-HSA stimulation in EV cells and further increased in HIF-1α-overexpressing cells. TAL significantly reduced IL-4 and IL-13 levels in EV control cells, but this suppression was markedly blunted in the overexpression group (Figures 6E,F).

In addition, treatment with YC-1 (20 μM), a pharmacological inhibitor of HIF-1α, significantly reduced HIF-1α protein expression in hypoxic model cells (Supplementary Figure S3), and decreased β-hex release as well as IL-4 and IL-13 secretion (Figures 6G–I). Notably, combined treatment with TAL and YC-1 produced a greater inhibition of both degranulation and cytokine production than either treatment alone. Collectively, these findings suggest that TAL attenuates mast cell activation and Th2 cytokine production at least partially through the regulation of HIF-1α.

## 4 Discussion

In this study, we demonstrate for the first time that the total alkaloids from *Leonurus* (TAL) exhibit protective effects in an OVA-induced murine model of allergic asthma, predominantly by inhibiting mast cell activation via the HIF-1α signaling pathway. This provides novel insight into the immunomodulatory potential of *L. japonicus*, a traditional herb historically used for gynecological disorders. Initially, TAL significantly ameliorated hallmark features of allergic asthma, including allergic asthma–like behavior, airway hyperresponsiveness, and peribronchial inflammation *in vivo*. Concomitantly, a substantial reduction in mast cell infiltration and degranulation was observed in lung tissues. These *in vivo* findings were corroborated by *in vitro* data showing that TAL effectively suppressed β-hex release in IgE-sensitized RBL-2H3 cells, confirming its capacity to inhibit mast cell activation. Notably, dexamethasone (DEX) did not significantly restore body weight in OVA-challenged mice. This may be attributable to the well-documented metabolic effects of systemic glucocorticoids, including alterations in protein turnover and energy balance, which can offset short-term weight recovery (Li and Cummins, 2022; Lockett et al., 2024). Importantly, body weight is not a primary efficacy endpoint in experimental asthma models, where airway inflammation and hyperresponsiveness are the principal indicators of therapeutic response. Importantly, mast cells are increasingly recognized as central effectors in allergic responses, particularly under conditions of local hypoxia (Gwon et al., 2023). Persistent airway inflammation promotes a hypoxic microenvironment, which in turn stabilizes HIF-1α and enhances mast cell survival, degranulation, and cytokine production (including IL-4 and IL-13), thereby amplifying Th2-mediated inflammation and airway remodeling (Zhang, et al., 2025; Zhong et al., 2022). This creates a vicious cycle of hypoxia and immune activation. However, despite the critical role of hypoxia and HIF-1α in allergic airway disease, few natural products have been shown to modulate this axis effectively. Our data now suggest that TAL may serve as a promising intervention to break this pathological loop by targeting hypoxia-induced mast cell activation.

To explore potential mechanisms, transcriptomic analysis revealed distinct gene expression profiles between TAL-treated and hypoxic mast cells, with enrichment changes primarily related to hypoxia-associated signaling. Among these, HIF-1α emerged as a key regulatory node, consistent with its well-recognized role in hypoxia-driven inflammation and immune dysregulation. These observations align with prior studies implicating HIF-1α in Th2 immune polarization, mast cell survival, and pro-inflammatory mediator production under hypoxic conditions. Protein-level validation in both OVA-induced asthmatic lungs and CoCl_2_-stimulated mast cells confirmed TAL’s ability to decrease HIF-1α expression and hinder its nuclear translocation, thereby dampening downstream transcriptional activity. This effect translated into reduced mast cell degranulation and cytokine release, highlighting a functional consequence of HIF-1α inhibition. To functionally validate the involvement of HIF-1α, we utilized both gain and loss-of-function strategies. Overexpression of HIF-1α in RBL-2H3 cells enhanced mast cell activation and attenuated the inhibitory effects of TAL. Conversely, pharmacological inhibition with YC-1 reproduced and even amplified TAL-mediated suppression of degranulation and Th2 cytokines. These results indicate that TAL alleviates allergic inflammation partly via modulation of HIF-1α–dependent pathways, reinforcing the centrality of HIF-1α in this process.

Moreover, our study contributes to the growing body of evidence linking traditional medicinal plants to hypoxia-related pathologies. In classical Chinese medicine theory, *Leonurus japonicus* is known for promoting blood circulation and removing blood stasis, a concept increasingly understood as modulation of hypoxia and microcirculation at the molecular level (Zhang, et al., 2021). Previous research has suggested its benefit in ischemic stroke, myocardial infarction, and other hypoxia-associated conditions (Shang, et al., 2014). Our findings extend these observations to allergic asthma, suggesting that regulation of hypoxia-responsive signaling may underlie part of its broader pharmacological profile. While hypoxia and HIF-1α have been widely studied in tumor biology and chronic inflammatory diseases, their role in mast cell-mediated allergic responses remains underexplored. This study reveals that modulating HIF-1α signaling not only alters inflammatory cytokine expression and metabolic adaptation but also directly affects mast cell degranulation, offering a mechanistic bridge between tissue microenvironmental stress and immune activation. By identifying HIF-1α as a functional regulator in allergic inflammation, our work provides mechanistic insight into hypoxia-associated immune regulation in asthma.

Despite these promising findings, several limitations exist. First, while the total alkaloid extract was standardized and its major components quantified via HPLC, the specific compounds responsible for the anti-asthmatic effects were not isolated or characterized functionally. Second, although the *in vitro* and *in vivo* findings are consistent, detailed pharmacokinetics and bioavailability data for TAL remain unaddressed. Lastly, the current study focused primarily on mast cells; whether other immune cell types are involved in TAL’s regulatory effects requires further clarification.

From an ethnopharmacological perspective, this study extends the traditional indications of *L. japonicus* into the respiratory domain, offering a scientific basis for its novel use in allergic diseases such as asthma. The suppression of hypoxia-driven mast cell activation positions TAL as a promising candidate for further development as a complementary therapy in allergic airway inflammation, especially in patients with severe or steroid-insensitive asthma where hypoxia plays a dominant pathological role.

In summary, this study demonstrates that total alkaloids from *L. japonicus* exert protective effects in allergic asthma by suppressing hypoxia-driven mast cell activation through modulation of HIF-1α signaling. By integrating *in vivo, in vitro,* and transcriptomic analyses, we identify HIF-1α as a functional regulator linking tissue hypoxia to mast cell–mediated Th2 inflammation. TAL not only attenuated airway inflammation and hyperresponsiveness but also disrupted the pathological hypoxia–immune activation loop. These findings provide mechanistic insight into the anti-asthmatic potential of TAL and support further investigation of HIF-1α–targeted strategies in allergic airway diseases.

## Data Availability

The RNA sequencing data have been deposited in the NCBI Sequence Read Archive (SRA) under BioProject accession number PRJNA1426373.
