# Supplementary material for: Total alkaloids of Leonurus alleviate allergic asthma and inflammation responses by inhibiting hypoxia-induced factor-1⍺-mediated mast cell activation
Source: Front Pharmacol. 2026 Mar 9;17:1783196. doi: 10.3389/fphar.2026.1783196 (PMC13006691; doi:10.3389/fphar.2026.1783196)
Supplement: Supplementary file 1 [file DataSheet1.docx]

Supplementary Material

## Supplementary Figures


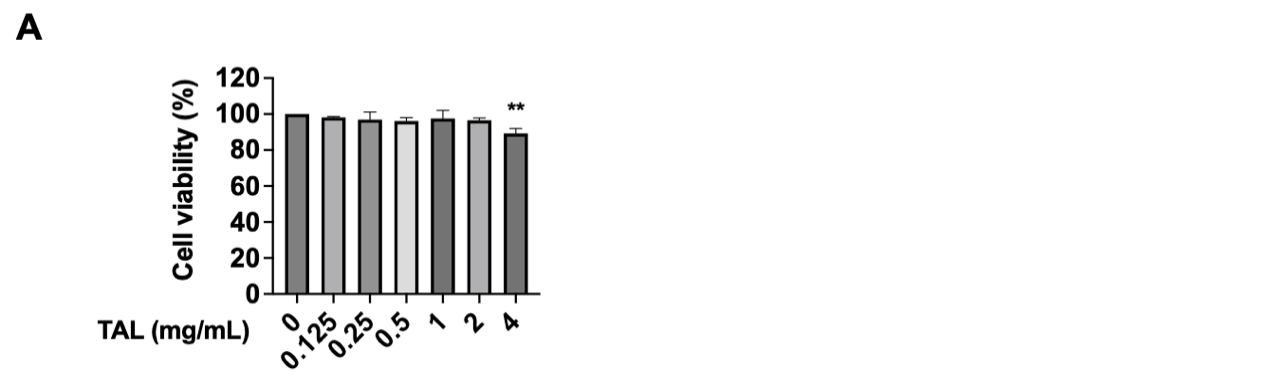


**Supplementary Figure 1.** CCK-8 assay assessing the cell viability of RBL-2H3 cells treated with TAL.


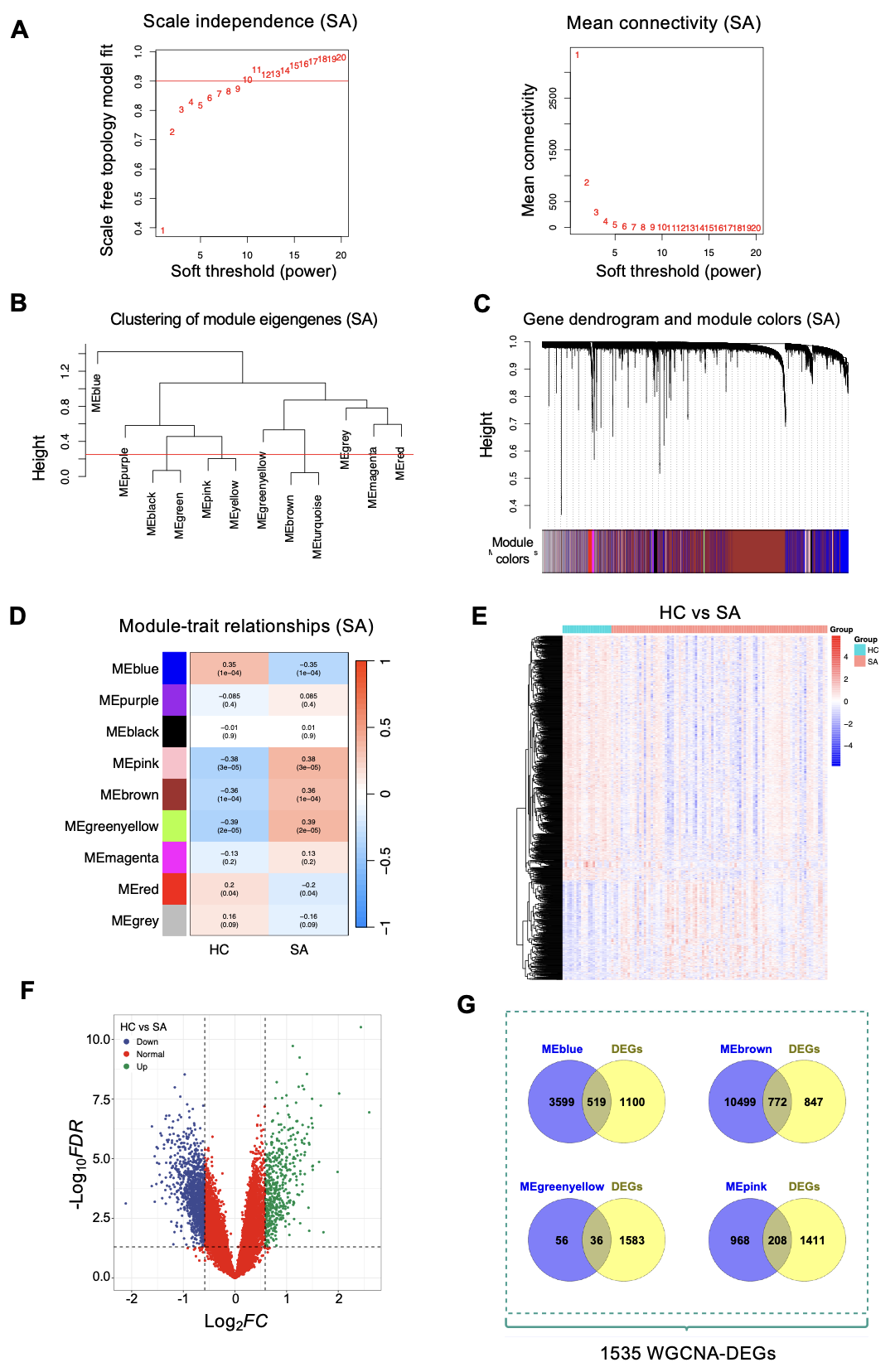


**Supplementary Figure 2.** Identification of severe asthma (SA)-associated genes by WGCNA and differential expression analysis. (A) Soft-thresholding power selection to achieve scale-free topology in WGCNA. (*β*=10) (B) Clustering dendrogram of module eigengenes in SA samples. (C) Gene dendrogram and module color assignment based on dynamic tree cutting. (D) Heatmap of module–trait relationships showing correlations between modules and clinical phenotypes. (E) Heatmap of differentially expressed genes (DEGs) between healthy controls (HC) and SA patients. (F) Volcano plot of DEGs between HC and SA. (G) Venn diagram showing the overlap between DEGs and WGCNA modules (MEblue, MEbrown, MEgreenyellow, MEpink). (H) Protein–protein interaction (PPI) network constructed from the intersected genes (WGCNA-DEGs).


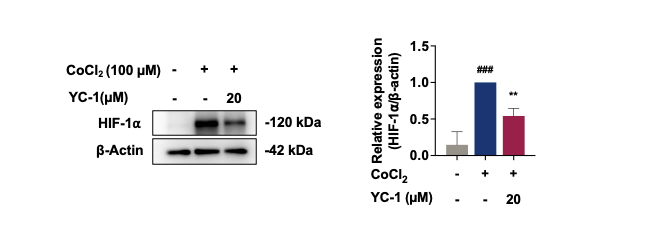


**Supplementary Figure 3.** Western blotting validation and quantification of YC-1 on HIF-1α protein expression in RBL-2H3 cells after CoCl_2_ treatment.
